# Prognostic Value of Body Weight-Independent Tricuspid Annular Plane Systolic Excursion to Systolic Pulmonary Arterial Pressure Ratio in Canine Precapillary Pulmonary Hypertension: A Retrospective Study

**DOI:** 10.3390/ani15233365

**Published:** 2025-11-21

**Authors:** Emilie Van Renterghem, Margaux Legrand, Marine Lekane, Elodie Roels, Kris Gommeren, Anne-Christine Merveille

**Affiliations:** Department of Clinical Sciences, Faculty of Veterinary Medicine, University of Liège, 4000 Liège, Belgium; emilie.vanrenterghem@uliege.be (E.V.R.);

**Keywords:** echocardiography, survival, right ventricular pulmonary artery coupling, tricuspid regurgitation, dogs

## Abstract

Background: The prognostic use of both the echocardiographic parameters tricuspid annular plane systolic excursion (TAPSE) and systolic pulmonary artery pressure (sPAP) in dogs with pulmonary hypertension (PH) has shown conflicting results. The ratio of TAPSE/sPAP, not yet described in dogs, is used for the evaluation and risk assessment in people with PH and is a validated surrogate for right ventricular to pulmonary artery coupling with lower values being associated with poor outcome. This study aimed to describe TAPSE/sPAP in dogs with precapillary PH (PCPH) due to various diseases. It demonstrates the correlation of this ratio with echocardiographic indices, its association with heart failure (HF) and its prognostic value. Methods: Medical records and echocardiographic data from 95 client-owned dogs with estimated mild (*n* = 10), moderate (*n* = 31) or severe (*n* = 54) PCPH were retrospectively reviewed. Body weight-independent TAPSE/sPAP ratios were obtained, and short-term and overall survival until death from all causes or cardio-pulmonary death (CPD) were assessed. Results: TAPSE/sPAP was lower in dogs with HF and correlated with other echocardiographic variables commonly altered in dogs with PCPH. Different TAPSE/sPAP ratios, such as nTAPSE/(sPAP(m/s)), were independent predictors of short-term CPD, as was having pulmonary thromboembolism. For overall survival only HF was a risk factor of CPD. The cut-off value of <1.05 for nTAPSE/(sPAP(m/s)) was associated with shorter survival for dogs dying from CPD. Conclusions: The TAPSE/sPAP ratio is a non-invasive echocardiographic parameter that provides prognostic information, especially in short-term survival, in dogs with PCPH and may aid in risk stratification.

## Simple Summary

Pulmonary hypertension can be caused by various diseases affecting the lung and/or pulmonary arteries. This condition increases the workload of the right ventricle and outcome of patients is heavily dependent on right ventricular function. The echocardiographic parameters tricuspid annular plane systolic excursion (TAPSE), which is commonly used to assess right ventricular systolic function, and estimated systolic pulmonary artery pressure (sPAP), derived from tricuspid regurgitation velocity, have both been studied in dogs with pulmonary hypertension. The prognostic use of these echocardiographic parameters separately in dogs with pulmonary hypertension shows conflicting results. The ratio of TAPSE/sPAP is used for evaluation and risk assessment in humans with pulmonary hypertension and describes how effectively the right ventricle contracts in response to increased afterload. This study describes the TAPSE/sPAP ratio in dogs with precapillary pulmonary hypertension and demonstrated that lower values are linked to heart failure and a higher risk of death. This suggests that the TAPSE/sPAP ratio could be a valuable non-invasive tool to help veterinarians assess prognosis in dogs with precapillary pulmonary hypertension.

## 1. Introduction

Pulmonary hypertension (PH) in dogs is a common entity with many underlying disease processes, classified in different groups depending on the cause: pulmonary arterial hypertension (group 1), left heart disease (group 2), secondary to respiratory disease/hypoxia (group 3), pulmonary emboli (group 4), parasitic disease (group 5) or multifactorial/unclear mechanisms (group 6) [1]. All of the aforementioned groups except for group 2—which is characterized by postcapillary PH due to elevated pulmonary venous pressure—contribute to precapillary PH (PCPH) through increased pulmonary vascular resistance, pulmonary overcirculation or a combination of these mechanisms [1]. Right heart catheterization is the gold standard for evaluating the presence and severity of PH and can be used to assess right ventricular function, with the latter being the main determinant of outcome in PH [2,3,4]. The right ventricle (RV) responds to pressure overload, with increased contractility and hypertrophy, to maintain cardiac output and RV-to-pulmonary arterial (RV-PA) coupling (homeometric adaptation). Heterometric adaptation occurs when the RV is exhausted, which will lead to RV dilation, increased filling pressure, decreased cardiac output (CO) and thus RV-PA decoupling, which can lead to heart failure (HF) [2,5]. To measure RV-PA coupling, right heart catheterization is necessary to perform pressure–volume loops and obtain the volume-loop-derived end-systolic/arterial elastance [Ees/Ea] ratio with Ees and Ea representing RV function and afterload, respectively [2].

In veterinary medicine, invasive measurements are rarely used for diagnosing PH or assessing the Ees/Ea ratio because of the need for general anesthesia and costs; instead, echocardiographic surrogates are preferred [1].

This is why guidelines were published to help evaluate the probability of having PH using echocardiography in dogs [1]. Tricuspid regurgitation (TR) is the main echocardiographic parameter to provide an estimate for systolic pulmonary artery pressure (sPAP) and the probability of PH in dogs [1]. The sPAP represents the systolic pressure within the pulmonary artery and serves as a direct echocardiographic estimate of pulmonary arterial pressure [1]. There is however conflicting evidence about sPAP and its prognostic value in dogs with PH, with some studies reporting a significant association between sPAP and clinical outcome, while others fail to demonstrate this [4,6,7]. The echocardiographic parameter TAPSE is commonly used for the assessment of an RV systolic function as it reflects the longitudinal shortening of the RV [8]. In veterinary medicine, TAPSE is normalized to body weight (BW) (and thus body weight-independent) given the variety of dog size and body conformation [4,8]. Decreased values of TAPSE are not consistently observed in dogs with varying severities of PH [9]. To date, only one study identified that a decreased value of normalized TAPSE, below 3.23 mm/kg^0.284^, was associated with increased risk of death in dogs with PH [4].

When evaluating estimated sPAP or TAPSE separately, there is a risk of underestimating disease severity. For example, sPAP may be underestimated in cases of severe RV dysfunction, while TAPSE can be overestimated in the presence of significant TR [2,4,10]. The TAPSE/sPAP ratio combines the RV length–tension relationship and has been validated as a non-invasive surrogate to assess RV-PA coupling in humans as it represents RV function (TAPSE) and afterload (sPAP) similarly to the Ees/Ea ratio [2]. It has been shown to predict outcomes in patients with both precapillary and postcapillary PH separately [3,5,11] and European (ESC/ERS) and American (ASE) guidelines recommend the TAPSE/sPAP ratio as a non-invasive marker for the evaluation and risk stratification of patients with PH [11,12].

To the authors’ knowledge, the ratio TAPSE/sPAP has not been described in dogs with PH. The authors chose to describe TAPSE/sPAP in dogs with PCPH, as postcapillary PH is predominantly associated with degenerative mitral valve disease. This condition induces right ventricular hyperkinesia, which can alter (and overestimate) TAPSE measurements and therefore influence the ratio [13,14]. The objectives of this study were to describe body weight-independent TAPSE/sPAP in dogs with probable PCPH and to investigate its correlation with various echocardiographic indices of right and left heart size and function. In addition, this study aimed to characterize this ratio in the presence of HF and assess whether it could serve as a prognostic factor. We hypothesize that this parameter will decrease in the presence of clinical signs of HF, with increased severity of PCPH, and can serve as a prognostic factor for survival.

## 2. Materials and Methods

### 2.1. Animals

Records of dogs between November 2014 and February 2023 at the University of Liège were retrospectively inspected for dogs of all breeds and ages with a diagnosis of PCPH based on the presence of an increased velocity of TR. Dogs with PCPH and concomitant degenerative mitral valve disease, that had quantitative measurements of the left ventricular internal dimensions in diastole and systole within the upper 97.5th percentile of healthy dogs [15] and a normal left atrial size (left atrium-to-aortic root ratio < 1.6) [16], were included if the mitral valve regurgitation was mild. Dogs that required sedation for echocardiography were not eligible for inclusion. Patients with congenital or hemodynamically acquired heart disease (other than degenerative mitral valve disease without left heart enlargement) or with a pathological arrhythmia were also not eligible for inclusion.

### 2.2. Clinical Data

Signalment (age, BW, breed) and cardiac medications administered prior to echocardiography (diuretics, sildenafil, pimobendan and angiotensin-converting enzyme inhibitors) were recorded.

Dogs were classified as having low CO failure if they had clinical signs compatible with syncope, pale mucosae, weak pulses, low systemic blood pressure and/or right-sided congestive failure (R-CHF) made evident by the sonographic appearance of abdominal/pleural/pericardial effusion with a subjectively enlarged vena cava with reduced compliance. The term ‘heart failure (HF)’ is used throughout this manuscript but when it is used alone, it is defined as low CO and/or R-CHF.

The patient’s record was investigated for further examinations, if available, such as complete blood count, serum chemistry, coagulation profile, blood pressure, thoracic radiographs with or without fluoroscopy, bronchoscopy, computed tomography of the thorax and parasitic testing for *Dirofiliaria* and *Angiostrongylus*. Dogs were then classified according to the underlying cause of PH, as previously described [1].

Overall survival, until death at any moment, and short-term survival (death < 31 days after echocardiography) were analyzed. Cause of death was obtained by reviewing the patient’s record or by telephoning the referring veterinarian or owner. Cardio-pulmonary death (CPD) was defined as death or euthanasia attributable to cardio-pulmonary disease (due to dyspnea/tachypnea, R-CHF or sudden death). If the cause of death was uncertain, the patient was classified as having no CPD.

### 2.3. Echocardiography

All echocardiographic studies investigated were previously performed in right and left recumbency and had 2D, M-mode, spectral and color Doppler performed as previously described [17] with images that were adequate to visualize right heart structures. Image acquisition was previously performed by a board-certified veterinary cardiologist or cardiology resident under guidance of a board-certified cardiologist (Vivid I and Vivid E95 (General Electric Medical System) equipped with phased-array probes with a frequency of, respectively, 2.2–3.5 and 5.5–7.5 MHz or 1.4–4.6, 2.4–8.0 and 4.0–12.0 MHz, and with a single lead electrocardiogram connected simultaneously, Waukesha, WI). All measurements were performed at least 3–5 times, on consecutive beats, by a board-certified cardiologist (A-C.M.). Table 1 summarizes most abbreviations used in the following paragraphs to facilitate reading.

A measurable, clearly visible TR jet was required, as this was necessary for the estimation of sPAP. Dogs were divided into mild (30–50 mmHg), moderate (51–75 mmHg) and severe (>75 mmHg) PCPH based on TR pressure gradient (TRPG) using the simplified Bernouilli equation: TRPG = 4 × TR(m/s)^2^ [18]. The terms ‘mild’, ‘moderate’ and ‘severe’ are based on estimated pressure gradients but for the simplification of the manuscript, these terms will be used without ‘estimated’.

The TR severity was subjectively quantified as being mild, moderate or severe depending on the color-flow and continuous wave Doppler, as previously described [19]. A small jet on the color Doppler and faint parabolic visible on continuous wave was labeled as mild TR. If there was an intermediate TR jet on the color Doppler and a dense parabolic TR jet signal on the continuous wave Doppler, this was considered moderate. When a very large jet was present on the color Doppler and a dense parabolic signal was seen on the continuous wave Doppler, this was labeled as severe TR [19].

TAPSE was measured from an M-mode recording by aligning the cursor parallel to the right ventricular free wall at the lateral aspect of the tricuspid valve annulus, with a parasternal long-axis view focused on the right ventricle [8]. In some cases, the anatomic M-mode technique was used on stored 2D images [20]. TAPSE was normalized to BW by the following formula: $nTAPSE=\frac{mm}{{kg}^{0.285}}$ [21] or by TAPSE/Ao [22].

Different combinations of body weight-independent TAPSE (nTAPSE and TAPSE/Ao) and estimates of sPAP (TRPG in m/s or mmHg) were then obtained. The ratio was multiplied by tenfold for (TAPSE/Ao)/sPAP(m/s) and nTAPSE/sPAP(mmHg) and by a hundredfold for (TAPSE/Ao)/sPAP(mmHg) to obtain numbers in the same decimals.

Other echocardiographic parameters of right heart remodeling were recorded. Right atrial diameter (RAD) was measured from a right parasternal 4-chamber view preceding tricuspid valve opening. Only the minor dimension, parallel to the tricuspid annulus, was measured and then normalized to BW: $nRAD=mm/kg^{0.30}$ [23]. Right ventricular internal diameter in diastole (RVDd) was measured using a right parasternal long-axis view parallel to the tricuspid valve with a view optimized for the right ventricle [23]. The diameter of the aortic root was measured in a right parasternal short-axis view parallel to the non- and left-coronary leaflets at the end of systole after aortic valve closure [16]. The ratio of RVDd on aortic root (RVDd/Ao) was obtained [24]. Right ventricular area was measured on a left apical view optimized for the right heart by tracing the endocardial border in end-diastole (RVAd) and in end-systole (RVAs) and was normalized to BW (nRVAd and nRVAs) by the following equations: $nRVAd={cm}^{2}/kg^{0.665}$ and $nRVAs={cm}^{2}/kg^{0.695}$, respectively, as previously described [21]. RV fractional area change (%) (RVFAC) of the right ventricle was calculated by the following formula: $\left[\frac{RVAd\;-\;RVAs}{RVAd}\right]\times 100$ [25].

Thickness of the parietal free walls of the right and left ventricle were obtained in a 2D right parasternal 4-chamber long-axis or short-axis view at end-diastole. The ratio of the right ventricular free wall in diastole to left ventricular free wall in diastole (RVFWd/LVFWd) was obtained [24].

The eccentricity index (EI) was obtained by measuring the minor axis of the left ventricle parallel to the septum divided by the minor axis perpendicular to the septum in a right parasternal short-axis view in diastole (EId) and systole (EIs), as previously described [24]. Left atrium-to-aortic root ratio (LA/Ao) was obtained in a right parasternal short-axis view [17].

The main pulmonary trunk was measured and indexed to the aorta (MPA/Ao) [26] as was the right pulmonary artery diameter, which was indexed to the right medial pulmonary vein diameter (PV/PA) [27].

### 2.4. Statistical Analysis

Data distribution were visually assessed and expressed as mean (±standard deviation) for normally distributed data or median (and interquartile range (IQR)) for non-normal distributed data.

Comparisons of quantitative parameters (age, weight, pressure gradient difference and TAPSE/sPAP) between PCPH severity subgroups were performed using ANOVA or simple linear regression as appropriate. The impact of PCPH severity on qualitative parameters was analyzed using univariate logistic regression. Comparisons of nTAPSE and TAPSE/Ao between PCPH severity subgroups were conducted with the Kruskal–Wallis test, applying a Bonferroni-corrected significance threshold of *p* < 0.0167. Differences in TAPSE/sPAP between dogs with and without clinical signs compatible with HF were assessed using the Mann–Whitney U test with a significance level of *p* < 0.05.

The correlation between TAPSE/sPAP and echocardiography measurements were compared with Spearman’s rank correlation and was defined as high when r was between 0.7 and 0.89, moderate when r was between 0.4 and 0.69 and weak when r was inferior to 0.39 [28].

Overall and short-term survival until death from all causes and until CPD were reported using Kaplan–Meier curves with a subanalysis that was performed by removing dogs with group 5 PCPH (parasitic disease). Cox proportional hazard models were built to determine which parameters were prognostic factors for the risk of death—from all causes or from CPD—for overall survival and short-term survival. Simple (=univariate) models were built and statistically significant variables (at <0.10 level) were included in multiple Cox model. Univariate and multivariate logistic regression models including age, etiology, PCPH severity, HF, and TAPSE/sPAP were tested as prognostic factors.

Since the four TAPSE/sPAP parameters are highly correlated and represent the same measure, different multiple models were built, including those parameters, one by one. Results were reported, as Hazard Ratios (HR), their 95% confidence intervals (95%CI) and *p*-values. All results were considered significant at the 5% level of uncertainty (*p* < 0.05). An exploratory analysis of TAPSE/sPAP was conducted using Kaplan–Meier survival curves and Wilcoxon test, with the ratio dichotomized at the median value.

Calculations were performed using SAS software (Version 9.4) and XLSTAT 2019.11.62918 (Addinsoft, Paris, France). Survival analysis and graphs were computed using R programming (Version 4.2.2) and XLSTAT.

## 3. Results

### 3.1. Study Population

In total, 95 dogs with mild (*n* = 10), moderate (*n =* 31) and severe (*n =* 54) PCPH were included in this study. In the mild PCPH group, West Highland White Terriers (*n =* 3), crossbreeds (*n* = 2) and five dogs of different breeds, each represented only once, were included. In the moderate PCPH group, there were West Highland White Terriers (*n* = 7), crossbreeds (*n* = 5), Chihuahuas (*n* = 5), Shih Tzus (*n* = 3), Jack Russel Terriers (*n* = 2), Bichon Frisé (*n* = 2), French Bulldogs (*n* = 2) and 5 dogs of different breeds, each represented only once, included. In the severe PCPH group, there were mainly Chihuahuas (*n =* 11), French Bulldogs (*n* = 6), Jack Russel Terriers (*n* = 5), Belgian Shepherds (*n* = 4), Pugs (*n* = 3), Shih Tzus (*n* = 3), crossbreeds (*n* = 3), Dachshunds (*n* = 2) and 20 dogs of different breeds, each represented only once, included.

BW and age were not significantly different between the three groups (*p* < 0.22; Table 2). Forty-nine dogs were in HF (thirty-nine with low CO, twenty-seven with R-CHF and eighteen with both). The frequency of having HF (low CO, R-CHF, or both) increased with severity of PCPH (*p* < 0.007; Table 2). Forty dogs were on treatment at the time of diagnosis, but the type and the proportion of dogs on cardiac treatment did not differ between the three groups of PCPH severity (*p* < 0.65; Table 2).

**Figure 1:**
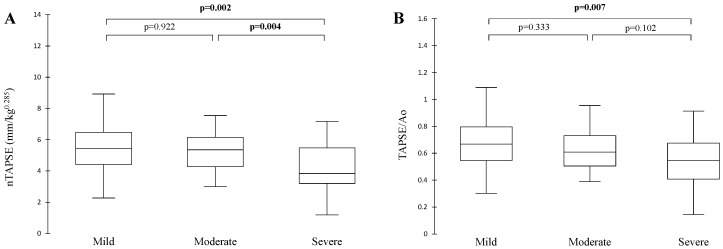
Box plots representing the comparison of nTAPSE (**A**) and TAPSE/Ao (**B**) between different severity subgroups of PCPH. These are demonstrating lower TAPSE measurements in severe compared to mild PCPH for both ratios and between moderate and severe PCPH for nTAPSE. Overlap is seen between all subgroups for both nTAPSE and TAPSE/Ao. Statistics performed by Kruskal–Wallis; significant *p*-values are in bold (<0.0167). Ao, aorta; nTAPSE, normalized tricuspid annular plane systolic excursion; PCPH, precapillary pulmonary hypertension; sPAP, systolic pulmonary arterial pressure.

Both nTAPSE and TAPSE/Ao were significantly decreased in dogs with severe PCPH compared to mild PCPH, but not between moderate and mild PCPH (*p* < 0.007; Figure 1). The parameter nTAPSE was also significantly decreased in severe compared to moderate PCPH (*p* < 0.004; Figure 1).

In this study, subjective TR severity was based on color and continuous wave Doppler and was assessed as mild in 29/95 dogs (30%), moderate in 33/95 dogs (35%) and severe in 33/95 dogs (35%). The prevalence of TR was significantly different in dogs with mild, moderate and severe PCPH (*p* < 0.0001; Table 2). An overview of these data can be found in Table 2. The etiologies of PCPH are presented in Table 3.

### 3.2. TAPSE/sPAP: Correlation with Different Echocardiographic Parameters and Association with HF

**Figure 2:**
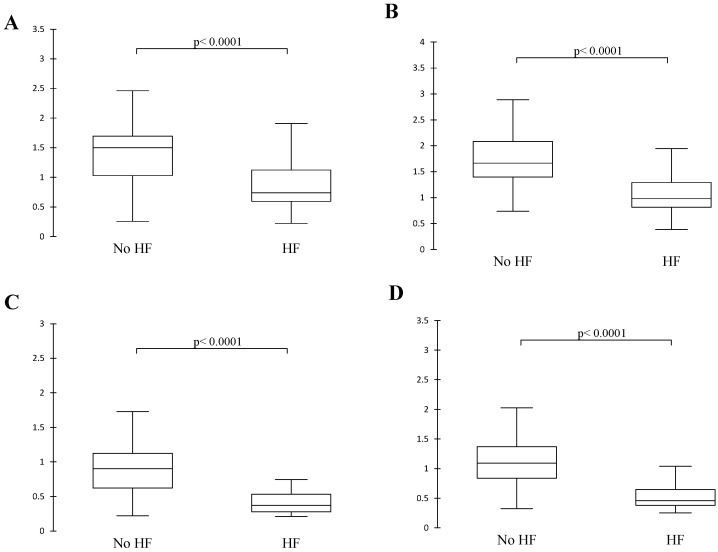
Box plots illustrating TAPSE/sPAP in dogs with or without HF. (**A**) nTAPSE/sPAP(m/s); (**B**) (TAPSE/Ao)/(sPAP(m/s)) × 10; (**C**) nTAPSE/(sPAP(mmHg)) × 10; (**D**) (TAPSE/Ao)/(sPAP(mmHg)) × 100. Statistics performed by Mann–Whitney U test with significant *p*-value < 0.05. Ao, aorta; HF, heart failure; nTAPSE, normalized tricuspid annular plane systolic excursion; sPAP, systolic pulmonary arterial pressure.

As expected, sPAP increased with group severity (Table 4). The four TAPSE/sPAP ratios decreased with increased severity (*p* < 0.0001; Table 4). All the echocardiographic parameters had a moderate-to-high correlation with the different TAPSE/sPAP (*p* < 0.001) ratios except for LA/Ao where the correlation was weak (*p* < 0.023; Table 5). The four TAPSE/sPAP ratios were significantly lower in dogs with HF (*p* < 0.0001; Figure 2).

### 3.3. Short-Term Survival Analysis

**Figure 3:**
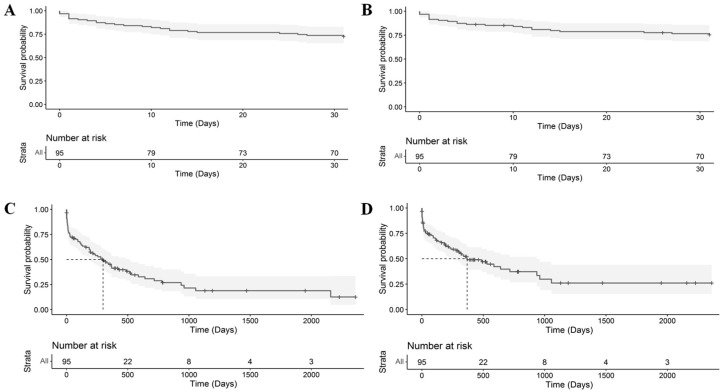
Kaplan–Meier curves demonstrating (**A**) short-term survival until death from all causes; (**B**) short-term survival until CPD; (**C**) overall survival until death from all causes with a median survival time of 279 days; (**D**) overall survival time until CPD with a median survival time of 365 days. CPD, cardio-pulmonary death.

In short-term, 26 dogs died of which 23 were from CPD (Figure 3) and three from causes unrelated to a cardio-pulmonary condition.

For short-term death from all causes only, (TAPSE/Ao)/(sPAP(m/s)) × 10 was a prognostic factor in the multivariate analysis (*p* < 0.03; Table 6).

Having thrombo-embolic disease (group 4 of PCPH) was associated with a threefold increased risk for short-term CPD (*p* < 0.041; Table 7). Also, nTAPSE/(sPAP(m/s)) and (TAPSE/Ao)/(sPAP(m/s)) × 10 were both independent risk factors for short-term CPD with the risk decreasing when the parameter increased (*p* < 0.044; Table 7).

### 3.4. Overall Survival Analysis

Dogs were followed for a median of 214 (IQR: 24–492) days. Overall mortality was 64, including 51 from CPD. Median survival time was 279 days for death from all causes and 365 days from CPD (Figure 3). Removing dogs with group 5 (parasitic) PCPH did not influence median survival times.

Dogs with thrombo-embolic disease had a twofold risk of death from all causes in the multiple Cox regression model (*p* < 0.03; Table 8).

**Figure 4:**
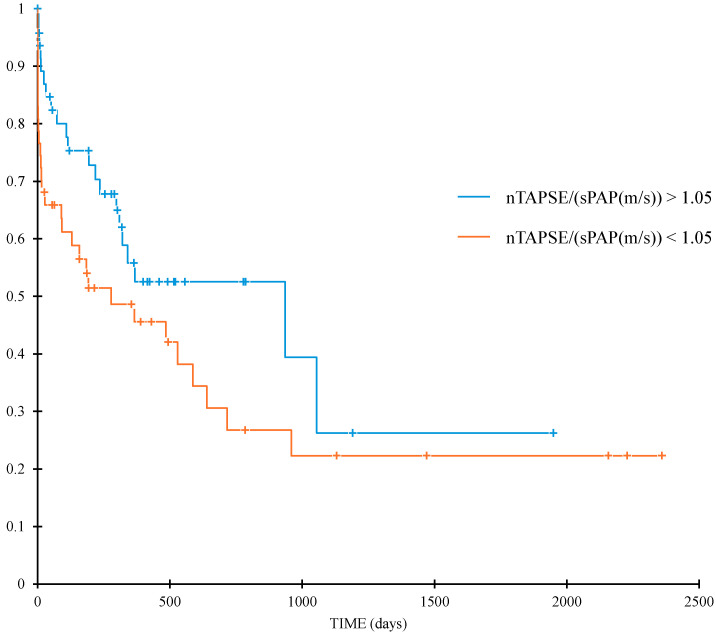
nTAPSE/(sPAP(m/s)) with a cut-off of >1.05 or <1.05 demonstrates a significant difference in median survival time for patients having CPD of 936 (95% CI: 309–NR) and 278 (95% CI: 90–640) days, respectively (*p* < 0.028). CPD, cardio-pulmonary death; NR, not reached; nTAPSE: normalized tricuspid annular plane systolic excursion; sPAP: systolic pulmonary arterial pressure.

The underlying etiology was not a risk factor for CPD (Table 9) but dogs with HF had a twofold increased risk of CPD (*p* < 0.03; Table 9). None of the TAPSE/sPAP ratios could be considered a risk factor for death from all causes or CPD. However, when nTAPSE/(sPAP(m/s)) was dichotomized according to the median value (1.05), it demonstrated a significant difference in median survival time, for survival until CPD, of 936 (95%CI:309–NR) and 278 (95%CI: 90–640) days for a cut-off value > 1.05 or <1.05, respectively (*p* < 0.028; Figure 4).

## 4. Discussion

This is the first study to describe body weight-independent ratios of TAPSE/sPAP in dogs with different severities of PCPH. This manuscript describes the significant correlation of TAPSE/sPAP with other echocardiographic indices, the association of lower values of this ratio with HF, and also the prognostic relevance of this ratio.

The benefit of TAPSE/sPAP is that it combines the RV length–tension relationship (longitudinal shortening with the ability of the RV to generate pulmonary pressure) and thus gives an idea about contractility and afterload at the same time [3]. It has emerged in human medicine as a non-invasive surrogate for RV–PA coupling and might perform better than TAPSE or sPAP alone [29]. Our results in dogs suggest, similarly, that lower TAPSE/sPAP values reflect impaired RV adaptation and are associated with adverse outcomes.

In cases of severe systolic dysfunction, the TR velocity will underestimate sPAP and might be inaccurate and misleading [4,10]. This is potentially why conflicting results/evidence has been reported regarding estimated sPAP and its prognostic value in dogs with PH [4,6,7]. In this study, the different PCPH severities did not seem to have an effect on long-term and short-term survival in the univariate and multivariate Cox models, indicating that severity of sPAP alone does not seem to affect survival.

The parameter TAPSE is a non-invasive echocardiographic parameter that correlates well with RV function, and its prognostic value has been demonstrated in humans [5] and also in dogs, only when dichotomized below a certain value [4]. With TAPSE, only RV function is taken into account, but not RV-PA coupling. Even though nTAPSE and TAPSE/Ao were significantly different between severe and mild PCPH, there was significant overlap between subgroups (Figure 2). In this study, TAPSE tended to decrease with disease severity, as previously demonstrated [4]. However, another study in dogs demonstrated that TAPSE was not significantly different among varying degrees of PH severity and between dogs with and without R-CHF [9]. TAPSE is preload dependent and might be altered by the presence of significant TR, often present in patients with PCPH [2]. Similarly to previous studies [4,9], we observed overlap for TAPSE measurements between all subgroups of PCPH. An explanation might be that many dogs in the moderate and severe subgroup had moderate to severe TR which might have altered TAPSE measurements. The interpretation of TAPSE might thus be difficult, as overestimation by significant TR might not reflect true RV contractile function and thus could compromise the reliability of TAPSE as a standalone marker of RV performance.

In human medicine, the TAPSE/sPAP ratio is expressed as millimeters per millimeter of mercury (mm/mmHg), since adult humans have relatively consistent body sizes and TAPSE values do not require normalization [30]. However, in dogs, TAPSE varies significantly with body weight [21]. Because this ratio had not previously been described in veterinary medicine, several body weight-independent forms of TAPSE, previously described, to sPAP (in m/s and mmHg) ratio were analyzed to see which ratio was best associated with outcome. As expected, TAPSE/sPAP decreased significantly with the severity of PCPH, given that the estimated TRPG (sPAP) increases between the subgroups. In human medicine, TAPSE/sPAP ratios obtained by echocardiography correlate with invasive measurements obtained by right heart catheterization, and the presence and severity of PH can be assessed in that way [2,11,31]. Invasive measurements were not performed in this study but future studies might evaluate the use of certain cut-offs of TAPSE/sPAP to aid in diagnosing probable PH in dogs, such as those suggested in the human guidelines [11,12].

All echocardiographic parameters evaluated in this study, besides LA/Ao, had moderate-to-high correlation with TAPSE/sPAP ratios. This seems to be in line with other studies that have demonstrated the effect of PH on echocardiographic parameters of right and left heart morphology and function [4,9,22,24,26,27,32]. This highlights the clinical relevance and consistency of TAPSE/sPAP with other established echocardiographic parameters of disease severity, which help in diagnosing canine PH [1].

In human medicine different studies describe the prognostic role of different TAPSE/sPAP cut-offs for different cardio-pulmonary diseases [3,31,33,34,35,36,37]. In some of these studies, TAPSE/sPAP is a significant prognostic factor for overall survival in multivariate Cox models which is not the case in this study [3,33,34,35]. Even though TAPSE/sPAP was not a prognostic factor in overall survival, when dichotomized below a certain cut-off value, TAPSE/(sPAP(m/s)) did demonstrate a significant difference on survival until CPD. This ratio is easy to obtain and might be of use in a clinical setting with the cut-off of <1.05 carrying worse prognosis. Dogs with PCPH in this study represented a heterogeneous population and the usefulness of TAPSE/sPAP as a prognostic parameter might be different depending on the underlying condition. Future studies investigating this ratio in dogs with different groups of PCPH could be interesting to determine its usefulness depending on the disease process causing PCPH and might show prognostic relevance, even in overall survival, such as in different disease processes in human medicine. Death due to euthanasia/financial restraints, incomplete or unclear documentation of the cause of death in patients surviving longer than one month and factors influencing either TAPSE or sPAP measurements might also have contributed to the absence of significance for overall survival. Additionally, the limited number of dogs may have contributed to the lack of statistical significance of this ratio as a risk factor for overall survival, as TAPSE/sPAP was associated with an increased risk of CPD in the overall survival analysis when dichotomized below a certain threshold.

The only risk factor for CPD in the overall survival analysis was the presence of HF. This is in agreement with a previous study that described that HF was a negative prognostic factor in dogs with PH [4]. Lower values of TAPSE/sPAP were associated with RV HF (Figure 2) which could be explained by decreased TAPSE measurements in RV dysfunction and more severe PH, both of which will lower the ratio [2]. In humans, decreased values of TAPSE/sPAP might predict future RV HF [38]. Lower TAPSE/sPAP values may indicate RV–PA uncoupling and could be useful for risk stratification and guiding treatment.

In the multivariable Cox models, pulmonary thromboembolism carried an increased hazard ratio for short-term CPD alongside different TAPSE/sPAP ratios. This might imply that TAPSE/sPAP is particularly useful for short-term risk stratification, as has been described in humans with pulmonary thromboembolism [29], because it reflects RV–PA coupling in the face of acute pulmonary vascular events. The fact that TAPSE/sPAP was an independent risk factor for short-term CPD likely reflects RV-PA uncoupling in more severe cases of PCPH, which is unsurprisingly associated with an increased risk of early mortality. Another possible reason for the observed association of TAPSE/sPAP with short-term rather than overall survival might be that data on clinical signs and the reason of death were more accessible in the patient’s record.

In this study, the majority of dogs (51 out of 64) died of CPD. This highlights the importance of identifying (echocardiographic) parameters such as TAPSE/sPAP, which could help stratify risk.

There are several limitations to this study. Firstly, the retrospective design of the study resulted in missing data and heterogeneous treatment approaches, both of which may have influenced patient outcomes. Dogs were thought to have CPD based on owner- reported symptoms or medical records of the dog before death. It is however possible that the reason for euthanasia or death was not caused by an underlying disease causing PCPH and that the cause of death might thus have been misclassified. In addition, some patients were euthanized due to poor prognosis or financial constraints, which may also have impacted the results, as these individuals might have survived longer if treatment had been pursued. All dogs in this study needed to have a TR to have an estimate of sPAP which makes that these data are only applicable to dogs having a measurable TR. Another limitation is that measurement of TAPSE might be underestimated when not perfectly aligned and could have contributed to underestimation of RV dysfunction. However, when the measurement was thought to be underestimated, the anatomic M-mode [20] was used to correct for this. In addition, an echocardiographic estimate of sPAP based on TRPG is influenced by acquisition and signal of the peak TR velocity. The TRPG can, for example, be overestimated in the presence of severe TR and underestimated due to improper Doppler beam orientation or incomplete acquisition of the TR jet profile [2,10,39,40].

The absence of right heart catheterization and invasive pressure measurements, which represent the gold standard for confirming PH, constitutes a limitation of this study. Guidelines have been established in veterinary medicine to help assess the probability of patients having low, intermediate or high PH based on TRPG and echocardiographic sites [1]. In this study, only TRPG was used to categorize dogs having PCPH and as a result, some patients may have been misclassified, and a proportion of those included might not have had true PCPH. However, dogs in the mild PCPH group had a median value of 44.9 mmHg as TRPG which approximates the 46 mmHg included in the guidelines to support dogs having intermediate probability of PH. Dogs in the moderate and severe PCPH group could at least be categorized as having intermediate probability of PH based on their TRPG [1].

## 5. Conclusions

In summary, in dogs with PCPH, TAPSE/sPAP correlated with other commonly altered echocardiographic variables, which reflects its clinical relevance. Dogs with HF had significantly lower TAPSE/sPAP, indicating that dogs with PCPH and lower values might be more at risk of demonstrating HF. Pulmonary thromboembolism and different TAPSE/sPAP ratios (nTAPSE/sPAP(m/s) and (TAPSE/Ao)/(sPAP(m/s)) × 10) were independently associated with short-term CPD which reflect its prognostic significance, especially in short-term survival. In the overall survival analysis, only HF was identified as a risk factor for CPD, whereas TAPSE/sPAP ratios were not. This may be due to the heterogeneity of the underlying causes of PCPH in this study population and warrants further investigation.

## Figures and Tables

**Table 1:** Abbreviations of echocardiographic parameters with full name and measurement type which might be altered and/or useful in the diagnosis of PH.

Parameter (Abbreviation)	Full Name	Measurement Type
sPAP (or TRPG)	Systolic pulmonary artery pressure (or tricuspid regurgitation pressure gradient)	Right ventricular pressure overload
nTAPSE	Tricuspid annular plane systolic excursion normalized to body weight	Right ventricular systolic function
TAPSE/Ao	Tricuspid annular plane systolic excursion normalized to aortic root ratio	Right ventricular systolic function
nRAD	Normalized right atrial diameter to body weight	Right atrial size
RVD/Ao	Right ventricular diameter to aortic root ratio	Right ventricular size
nRVAd	Normalized right ventricular area in diastole to body weight	Right ventricular size
nRVAs	Normalized right ventricular area in systole to body weight	Right ventricular systolic function
RVFAC	Right ventricular fractional area change	Right ventricular systolic function
RVFWd/LVFWd	Right ventricular free wall to left ventricular free wall ratio in diastole	Right ventricular free wall thickness
EI	Eccentricity index	Interventricular septal flattening
MPA/Ao	Mean pulmonary artery to aortic root ratio	Size of pulmonary artery
PV/PA	Pulmonary vein to pulmonary artery ratio	Size of pulmonary artery

PH, pulmonary hypertension.

**Table 2:** Demographic/clinical parameters and TR severity in function of severity of PCPH.

	Severity of PCPH			
	Mild	Moderate	Severe	*p*-Value
Age (years)	10.5 ± 3.0	10.6 ± 3.5	8.8 ± 4.4	0.10 ^(a)^
BW (kg)	13.6 ± 12.7	8.8 ± 5.6	11.2 ± 8.7	0.22 ^(a)^
Clinical signs of low CO (*n*)	1	7	31	<0.001 ^(b)^
Clinical signs of R-CHF (*n*)	2	2	23	0.007 ^(b)^
R-CHF and/or low CO (*n*)	2	7	39	<0.0001 ^(b)^
On cardiac treatment at presentation (*n*)	5	10	25	0.65 ^(b)^
Death (yes:no) (*n*)	6:4	21:10	37:17	
Long-term CPD: short-term CPD (*n*)	3:1	9:6	16:16	
TR severity (*n*) (mild:moderate:severe)	8:2:0	16:12:3	9:15:30	<0.0001 ^(b)^

^(a)^ ANOVA, ^(b)^ logistic regression, as appropriate. Data are expressed as median ± standard deviation or by numbers (*n*). BW, body weight; CO, cardiac output; CPD, cardio-pulmonary death; kg, kilogram; n, number; R-CHF, right-sided congestive failure; gradient; TR, tricuspid regurgitation.

**Table 3:** Number of dogs in function of etiology and severity of precapillary pulmonary hypertension (PCPH).

	Severity of PCPH		
	Mild	Moderate	Severe
Total number of dogs	10	31	54
Group 1 (pulmonary arterial hypertension)	0	0	1
Group 3 (pulmonary causes)	4	11	5
Group 4 (pulmonary thromboembolism)	1	3	11
Group 5 (parasitic)	0	3	17
Group 6 (multifactorial/unclear)	5	14	4

**Table 4:** TAPSE/sPAP in function of severity of PCPH.

	Severity of PCPH			
	Mild (*n =* 10)	Moderate (*n =* 34)	Severe (*n =* 54)	*p*-Value
sPAP (mmHg) based on TRPG	44.9 ± 5.52	61.2 ± 7.54	110.6 ± 28	<0.0001
nTAPSE/(sPAP (m/s))	1.8 ± 0.46	1.5 ± 0.53	0.84 ± 0.35	<0.0001
(TAPSE/Ao)/(sPAP (m/s)) × 10	2.1 ± 0.60	1.8 ± 0.58	1.1 ± 0.40	<0.0001
nTAPSE/(sPAP(mmHg)) × 10	1.3 ± 0.43	0.96 ± 0.34	0.44 ± 0.19	<0.0001
(TAPSE/Ao)/(sPAP(mmHg)) × 100	1.6 ± 0.55	1.2 ± 0.40	0.55 ± 0.24	<0.0001

Data are expressed as mean and standard deviation. Ao, aorta; nTAPSE, normalized tricuspid annular plane systolic excursion; PCPH, precapillary pulmonary hypertension; sPAP, systolic pulmonary artery pressure; TRPG, tricuspid regurgitation pressure gradient.

**Table 5:** Correlations of TAPSE/sPAP on echocardiographic parameters in dogs with PCPH.

	nTAPSE/(sPAP(m/s))	(TAPSE/Ao)/(sPAP(m/s)) × 10	nTAPSE/(sPAP(mmHg)) × 10	(TAPSE/Ao)/(sPAP(mmHg)) × 100	*p*-Value
sPAP based on TRPG (mmHg)	−0.74	−0.76	−0.86	−0.86	<0.001
nRAD (mm/kg^0.30^)	−0.58	−0.59	−0.68	−0.66	<0.001
RVDd/Ao	−0.60	−0.57	−0.71	−0.63	<0.001
nRVAd (cm^2^/kg^0.665^)	−0.48	−0.51	−0.55	−0.57	<0.001
RVFWd/LVFWd	−0.50	−0.47	−0.55	−0.51	<0.001
EId	−0.61	−0.62	−0.71	−0.69	<0.001
EIs	−0.74	−0.75	−0.82	−0.80	<0.001
LA/Ao	0.25	0.33	0.23	0.27	<0.023
MPA/Ao	−0.53	−0.46	−0.60	−0.52	<0.001
PV/PA	0.66	0.67	0.69	0.68	<0.001
nRVAs (cm_2_/kg^0.695^)	−0.65	−0.67	−0.68	−0.69	<0.001
RVFAC	0.61	0.64	0.59	0.60	<0.001
nTAPSE (mm/kg^0.285^)	0.77	0.81	0.72	0.70	<0.001
TAPSE/Ao	0.71	0.81	0.65	0.68	<0.001

Ao, aorta; EId, eccentricity index in diastole; EIs, eccentricity index in systole; LA/Ao, left atrium-to-aortic root; nRAD, normalized right atrial diameter, nRVAd, normalized right ventricular area in diastole; nRVAs, normalized right ventricular area in systole; nTAPSE, normalized tricuspid annular plane systolic excursion; PCPH, precapillary pulmonary hypertension; RVFAC, right ventricular fractional area change; RVFWd, right ventricular free wall in diastole; RVDd, right ventricular internal diameter in diastole; sPAP, systolic pulmonary artery pressure; TRPG, tricuspid regurgitation pressure gradient.

**Table 6:** Clinical and echocardiographic parameters as prognostic factors for the risk of short-term survival. Cox regression models for the risk of death from all causes in dogs with PCPH. Results are reported, as Hazard Ratios (HR) and their 95% confidence intervals (95%CI). Significant *p*-value for the simple Cox regression at <0.10 level and for multiple Cox regression model at <0.05. Bold values indicate statistical significance.

	Simple Cox Regression Models		Multiple Cox Regression Model 1		Multiple Cox Regression Model 2	
	HR (95%CI)	*p*-Value	Adj. HR (95%CI)	*p*-Value	Adj. HR (95%CI)	*p*-Value
Age (years)	1.0 (0.99–1.1)	0.99	-	-	-	-
Clinical signs of HF	1.5 (0.69–3.3)	0.30	-	-	-	-
Etiology of PCPH (ref. = group 6)						
Group 1	-	-	-	-	-	-
Group 3	1.1 (0.38–3.4)	0.83	1.5 (0.48–4.7)	0.49	1.6 (0.50–5.1)	0.43
Group 4	**2.4 (0.90–6.6)**	**0.08**	2.3 (0.87–6.3)	0.09	2.5 (0.93–6.9)	0.07
Group 5	1.2 (0.41–3.7)	0.71	0.93 (0.31–2.8)	0.90	0.92 (0.31–2.8)	0.88
Severity of PCPH (ref.= mild)						
moderate	1.1 (0.23–5.4)	0.89	-	-	-	-
severe	1.7 (0.39–7.3)	0.48	-	-	-	-
nTAPSE/(sPAP(m/s))	0.49 (0.23–11.1)	**0.08**	0.44 (0.19–1.0)	0.07	-	-
(TAPSE/Ao)/(sPAP(m/s)) × 10	**0.46 (0.22–0.98)**	**0.04**	-	-	**0.39 (0.16–0.91)**	**0.03**
nTAPSE/(sPAP(mmHg)) × 10	0.42 (0.14–1.3)	0.12	-	-	-	-
(TAPSE/Ao)/(sPAP(mmHg)) × 100	0.50 (0.20–1.2)	0.13	-	-	-	-

Ao, aorta; HF, heart failure; nTAPSE, normalized tricuspid annular plane systolic excursion; PCPH, precapillary pulmonary hypertension; sPAP, systolic pulmonary artery pressure.

**Table 7:** Clinical and echocardiographic parameters as prognostic factors for the risk of short-term survival. Cox regression models for the risk of CPD in dogs with PCPH. Results are reported, as Hazard Ratios (HR) and their 95% confidence intervals (95% CI). Significant *p*-value for the simple Cox regression at <0.10 level and for multiple Cox regression model at <0.05. Bold values indicate statistical significance.

	Simple Cox Regression Models		Multiple Cox Regression Model 1		Multiple Cox Regression Model 2		Multiple Cox Regression Model 3		Multiple Cox Regression Model 4	
	HR (95%CI)	*p*-Value	Adj. HR (95%CI)	*p*-Value	Adj. HR (95%CI)	*p*-Value	Adj. HR (95%CI)	*p*-Value	Adj. HR (95%CI)	*p*-Value
Age (years)	0.99 (0.89–1.1)	0.82	-	-	-	-	-	-	-	-
Clinical signs of HF	1.7 (0.74–3.9)	0.21	-	-	-	-	-	-	-	-
Etiology of PCPH (ref. = group 6)										
Group1	-	-	-	-	-	-	-	-	-	-
Group 3	1.2 (0.34–4.0)	0.82	1.7 (0.45–6.0)	0.45	1.8 (0.48–6.6)	0.39	1.7 (0.46–6.3)	0.42	1.6 (0.43–5.9)	0.48
Group 4	**3.1 (1.1–8.9)**	**0.03**	**3.0 (1.05–8.6)**	**0.041**	**3.3 (1.1–9.6)**	**0.027**	**3.2 (1.1–9.2)**	**0.032**	**3.1 (1.1–8.8)**	**0.037**
Group 5	1.6 (0.50–5.0)	0.44	1.2 (0.37–3.7)	0.80	1.2 (0.37–3.7)	0.79	1.1 (0.36–3.6)	0.83	1.1 (0.36–3.6)	0.83
Severity of PCPH (ref. = mild)										
moderate	1.9 (0.23–16.0)	0.54	-	-	-	-	-	-	-	-
sever	3.2 (0.42–24.1)	0.26	-	-	-	-	-	-	-	-
nTAPSE/sPAP(m/s):	**0.41 (0.17–0.96)**	**0.039**	**0.38 (0.15–0.97)**	**0.044**	-	-	-	-	-	-
(TAPSE/Ao)/(sPAP(m/s)) × 10	**0.40 (0.18–0.92)**	**0.031**	-	-	**0.33 (0.12–0.87)**	**0.025**	-	-	-	-
nTAPSE/(sPAP(mmHg)) × 10	**0.30 (0.083–1.1)**	**0.061**	-	-	-	-	0.23 (0.049–1.04)	0.056	-	-
(TAPSE/Ao)/(sPAP(mmHg × 100	**0.40 (0.14–1.1)**	**0.082**	-	-	-	-	-	-	0.33 (0.097–1.1)	0.081

Ao, aorta; CPD, cardio-pulmonary death; HF, heart failure; nTAPSE, normalized tricuspid annular plane systolic excursion; PCPH, precapillary pulmonary hypertension; sPAP, systolic pulmonary artery pressure.

**Table 8:** Clinical and echocardiographic parameters as prognostic factors for overall survival. Cox regression models for the risk of death from all causes in dogs with PCPH. Results are reported, as Hazard Ratios (HR) and their 95% confidence intervals (95%CI). Significant *p*-value for the simple Cox regression at <0.10 level and for multiple Cox regression model at <0.05. Bold values indicate statistical significance.

	Simple Cox Regression Models		Multiple Cox Regression Model	
	HR (95%CI)	*p*-Value	Adj. HR (95%CI)	*p*-Value
Age (years)	**1.1 (1.03–1.2)**	**0.004**	1.1 (0.99–1.1)	0.058
Clinical signs of HF	1.4 (0.84–2.3)	0.20	-	-
Etiology of PCPH (ref. = group 6)				
Group 1	-	-	-	-
Group 3	0.93 (0.48–1.8)	0.83	0.89 (0.46–1.7)	0.74
Group 4	**2.3 (1.2–4.4)**	**0.01**	**2.0 (1.05–3.8)**	**0.03**
Group 5	**0.50 (0.23–1.1)**	**0.09**	0.61 (0.27–1.4)	0.24
Severity of PCPH (ref. = mild)				
moderate	1.6 (0.63–4.0)	0.32	-	-
severe	1.4 (0.59–3.3)	0.45	-	-
nTAPSE/(sPAP(m/s))	0.99 (0.62–1.6)	0.95	-	-
(TAPSE/Ao)/(sPAP(m/s)) × 10	0.95 (0.62–1.5)	0.80	-	-
nTAPSE/(sPAP(mmHg)) × 10	0.96 (0.51–1.8)	0.89	-	-
(TAPSE/Ao)/(sPAP(mmHg)) × 100	0.99 (0.59–1.7)	0.98	-	-

Ao, aorta; HF, heart failure; nTAPSE, normalized tricuspid annular plane systolic excursion; PCPH, precapillary pulmonary hypertension; sPAP, systolic pulmonary artery pressure.

**Table 9:** Clinical and echocardiographic parameters as prognostic factors for overall survival. Cox regression models for the risk of CPD in dogs with PCPH. Results are reported, as Hazard Ratios (HR) and their 95% confidence intervals (95%CI). Significant *p*-value for the simple Cox regression at <0.10 level and for multiple Cox regression model at <0.05. Bold values indicate statistical significance.

	Simple Cox Regression Models		Multiple Cox Regression Model	
	HR (95%CI)	*p*-Value	Adj. HR (95%CI)	*p*-Value
Age (years)	**1.1 (1.0–1.2)**	**0.05**	1.1 (0.98–1.1)	0.15
Clinical signs of HF	**1.9 (1.1–3.4)**	**0.03**	**2.0 (1.1–3.9)**	**0.03**
Etiology of PCPH (ref. = group 6)				
Group 1	-	-	-	-
Group 3	0.95 (0.44–2.0)	0.90	1.1 (0.50–2.4)	0.80
Group 4	**2.5 (1.2–5.1)**	**0.01**	1.8 (0.86–3.8)	0.12
Group 5	0.68 (0.29–1.6)	0.36	0.62 (0.25–1.5)	0.30
Severity of PCPH (ref. = mild)				
moderate	1.7 (0.54–5.0)	0.38	-	-
severe	1.8 (0.64–5.2)	0.26	-	-
nTAPSE/(sPAP(m/s))	0.71 (0.42–1.2)	0.20	-	-
(TAPSE/Ao)/(sPAP(m/s)) × 10	0.73 (0.44–1.2)	0.23	-	-
nTAPSE/(sPAP(mmHg)) × 10	0.61 (0.29–1.3)	0.20	-	-
(TAPSE/Ao)/(sPAP(mmHg)) × 100	0.73 (0.40–1.4)	0.32	-	-

Ao, aorta; CPD, cardio-pulmonary death; HF, heart failure; nTAPSE, normalized tricuspid annular plane systolic excursion; PCPH, precapillary pulmonary hypertension; sPAP, systolic pulmonary artery pressure.

## Data Availability

The raw data supporting the conclusions of this article will be made available by the authors on request.

## Abbreviations

The following abbreviations are used in this manuscript:

Ao	Aortic root
ASE	American Society of Echocardiography
BW	Body weight
CO	Cardiac output
CPD	Cardio-pulmonary death
ECG	Electrocardiogram
EI	Eccentricity index
EId	Eccentricity index in diastole
EIs	Eccentricity index in systole
[Ees/Ea]	End-systolic/arterial elastance
ESC/ERS	European Society of Cardiology/European Respiratory Society
HF	Heart failure
HR	Hazard ratio
LA/Ao	Left atrium to aortic root ratio
LVFWd	Left ventricular free wall in diastole
MPA	Main pulmonary artery
nRAD	normalized right atrial diameter
nRVAd	normalized right ventricular end-diastolic area
nRVAs	normalized right ventricular end-systolic area
nTAPSE	normalized tricuspid annular plane systolic excursion
PCPH	Precapillary pulmonary hypertension
PH	Pulmonary hypertension
PV/PA	Pulmonary vein to pulmonary artery ratio
R-CHF	Right-sided congestive failure
RAD	Right atrial diameter
RV	Right ventricle/ventricular
RV-PA	Right ventricle to pulmonary artery
RVFAC	Right ventricular fractional area change
RVFWd	Right ventricular free wall in diastole
RVDd	Right ventricular internal diameter in diastole
sPAP	Systolic pulmonary artery pressure
TAPSE	Tricuspid annular plane systolic excursion
TAPSE/Ao	TAPSE normalized to aortic root
TAPSE/sPAP	TAPSE to sPAP ratio
TR	Tricuspid regurgitation
TRPG	Tricuspid regurgitation pressure gradient

## References

[1] Reinero C., Visser L.C., Kellihan H.B., Masseau I., Rozanski E., Clercx C., Williams K., Abbott J., Borgarelli M., Scansen B.A. (2020). ACVIM consensus statement guidelines for the diagnosis, classification, treatment, and monitoring of pulmonary hypertension in dogs. J. Vet. Intern. Med..

[2] Tello K., Wan J., Dalmer A., Vanderpool R., Ghofrani H.A., Naeije R., Roller F., Mohajerani E., Seeger W., Herberg U. (2019). Validation of the Tricuspid Annular Plane Systolic Excursion/Systolic Pulmonary Artery Pressure Ratio for the Assessment of Right Ventricular-Arterial Coupling in Severe Pulmonary Hypertension. Circ. Cardiovasc. Imaging.

[3] Tello K., Axmann J., Ghofrani H.A., Naeije R., Narcin N., Rieth A., Seeger W., Gall H., Richter M.J. (2018). Relevance of the TAPSE/PASP ratio in pulmonary arterial hypertension. Int. J. Cardiol..

[4] Visser L.C., Wood J.E., Johnson L.R. (2020). Survival characteristics and prognostic importance of echocardiographic measurements of right heart size and function in dogs with pulmonary hypertension. J. Vet. Intern. Med..

[5] Fauvel C., Raitiere O., Boucly A., De Groote P., Renard S., Bertona J., Lamblin N., Artaud-Macari E., Viacroze C., Schleifer D. (2022). Interest of TAPSE/sPAP ratio for noninvasive pulmonary arterial hypertension risk assessment. J. Heart Lung Transplant..

[6] Chan I.P., Weng M.C., Hsueh T., Lin Y.C., Lin S.L. (2019). Prognostic value of right pulmonary artery distensibility in dogs with pulmonary hypertension. J. Vet. Sci..

[7] Jaffey J.A., Wiggen K., Leach S.B., Masseau I., Girens R.E., Reinero C.R. (2019). Pulmonary hypertension secondary to respiratory disease and/or hypoxia in dogs: Clinical features, diagnostic testing and survival. Vet. J..

[8] Pariaut R., Saelinger C., Strickland K.N., Beaufrere H., Reynolds C.A., Vila J. (2012). Tricuspid annular plane systolic excursion (TAPSE) in dogs: Reference values and impact of pulmonary hypertension. J. Vet. Intern. Med..

[9] Vezzosi T., Domenech O., Costa G., Marchesotti F., Venco L., Zini E., Del Palacio M.J.F., Tognetti R. (2018). Echocardiographic evaluation of the right ventricular dimension and systolic function in dogs with pulmonary hypertension. J. Vet. Intern. Med..

[10] Soydan L.C., Kellihan H.B., Bates M.L., Stepien R.L., Consigny D.W., Bellofiore A., Francois C.J., Chesler N.C. (2015). Accuracy of Doppler echocardiographic estimates of pulmonary artery pressures in a canine model of pulmonary hypertension. J. Vet. Cardiol..

[11] Humbert M., Kovacs G., Hoeper M.M., Badagliacca R., Berger R.M.F., Brida M., Carlsen J., Coats A.J.S., Escribano-Subias P., Ferrari P. (2022). 2022 ESC/ERS Guidelines for the diagnosis and treatment of pulmonary hypertension. Eur. Heart J..

[12] Mukherjee M., Rudski L.G., Addetia K., Afilalo J., D’Alto M., Freed B.H., Friend L.B., Gargani L., Grapsa J., Hassoun P.M. (2025). Guidelines for the Echocardiographic Assessment of the Right Heart in Adults and Special Considerations in Pulmonary Hypertension: Recommendations from the American Society of Echocardiography. J. Am. Soc. Echocardiogr..

[13] Yuchi Y., Suzuki R., Teshima T., Matsumoto H., Koyama H. (2021). Utility of tricuspid annular plane systolic excursion normalized by right ventricular size indices in dogs with postcapillary pulmonary hypertension. J. Vet. Intern. Med..

[14] Poser H., Berlanda M., Monacolli M., Contiero B., Coltro A., Guglielmini C. (2017). Tricuspid annular plane systolic excursion in dogs with myxomatous mitral valve disease with and without pulmonary hypertension. J. Vet. Cardiol..

[15] Cornell C.C., Kittleson M.D., Della Torre P., Haggstrom J., Lombard C.W., Pedersen H.D., Vollmar A., Wey A. (2004). Allometric scaling of M-mode cardiac measurements in normal adult dogs. J. Vet. Intern. Med..

[16] Rishniw M., Erb H.N. (2000). Evaluation of four 2-dimensional echocardiographic methods of assessing left atrial size in dogs. J. Vet. Intern. Med..

[17] Thomas W.P., Gaber C.E., Jacobs G.J., Kaplan P.M., Lombard C.W., Moise N.S., Moses B.L. (1993). Recommendations for standards in transthoracic two-dimensional echocardiography in the dog and cat. Echocardiography Committee of the Specialty of Cardiology, American College of Veterinary Internal Medicine. J. Vet. Intern. Med..

[18] Kellihan H.B., Stepien R.L. (2010). Pulmonary hypertension in dogs: Diagnosis and therapy. Vet. Clin. North. Am. Small Anim. Pract..

[19] Lancellotti P., Moura L., Pierard L.A., Agricola E., Popescu B.A., Tribouilloy C., Hagendorff A., Monin J.L., Badano L., Zamorano J.L. (2010). European Association of Echocardiography recommendations for the assessment of valvular regurgitation. Part 2: Mitral and tricuspid regurgitation (native valve disease). Eur. J. Echocardiogr..

[20] Oyama M.A., Sisson D.D. (2005). Assessment of cardiac chamber size using anatomic M-mode. Vet. Radiol. Ultrasound.

[21] Feldhutter E.K., Domenech O., Vezzosi T., Tognetti R., Sauter N., Bauer A., Eberhard J., Friederich J., Wess G. (2022). Echocardiographic reference intervals for right ventricular indices, including 3-dimensional volume and 2-dimensional strain measurements in healthy dogs. J. Vet. Intern. Med..

[22] Caivano D., Dickson D., Pariaut R., Stillman M., Rishniw M. (2018). Tricuspid annular plane systolic excursion-to-aortic ratio provides a bodyweight-independent measure of right ventricular systolic function in dogs. J. Vet. Cardiol..

[23] Gentile-Solomon J.M., Abbott J.A. (2016). Conventional echocardiographic assessment of the canine right heart: Reference intervals and repeatability. J. Vet. Cardiol..

[24] Lekane M., Burnotte P., Gommeren K., Mc Entee K., Merveille A.C. (2024). Left ventricular eccentricity index to assess precapillary pulmonary hypertension in dogs. J. Vet. Cardiol..

[25] Visser L.C., Scansen B.A., Schober K.E., Bonagura J.D. (2015). Echocardiographic assessment of right ventricular systolic function in conscious healthy dogs: Repeatability and reference intervals. J. Vet. Cardiol..

[26] Grosso G., Tognetti R., Domenech O., Marchesotti F., Patata V., Vezzosi T. (2023). Echocardiographic reference intervals of the dimensions of the main pulmonary artery and the right pulmonary artery: A prospective study in 269 healthy dogs. J. Vet. Cardiol..

[27] Roels E., Merveille A.C., Moyse E., Gomart S., Clercx C., Mc Entee K. (2019). Diagnostic value of the pulmonary vein-to-right pulmonary artery ratio in dogs with pulmonary hypertension of precapillary origin. J. Vet. Cardiol..

[28] Schober P., Boer C., Schwarte L.A. (2018). Correlation Coefficients: Appropriate Use and Interpretation. Anesth. Analg..

[29] Kultursay B., Keskin B., Tanyeri S., Kulahcioglu S., Hakgor A., Mutlu D., Bulus C., Tokgoz H.C., Yucel E., Sekban A. (2024). Prognostic Impact of the Tricuspid Annular Plane Systolic Excursion/Pulmonary Arterial Systolic Pressure Ratio in Acute Pulmonary Embolism. Anatol. J. Cardiol..

[30] Kjaergaard J., Iversen K.K., Akkan D., Moller J.E., Kober L.V., Torp-Pedersen C., Hassager C. (2009). Predictors of right ventricular function as measured by tricuspid annular plane systolic excursion in heart failure. Cardiovasc. Ultrasound.

[31] Kazimierczyk R., Kazimierczyk E., Knapp M., Sobkowicz B., Malek L.A., Blaszczak P., Ptaszynska-Kopczynska K., Grzywna R., Kaminski K.A. (2021). Echocardiographic Assessment of Right Ventricular-Arterial Coupling in Predicting Prognosis of Pulmonary Arterial Hypertension Patients. J. Clin. Med..

[32] Feldhutter E.K., Domenech O., Vezzosi T., Tognetti R., Eberhard J., Friederich J., Wess G. (2022). Right ventricular size and function evaluated by various echocardiographic indices in dogs with pulmonary hypertension. J. Vet. Intern. Med..

[33] Legris V., Thibault B., Dupuis J., White M., Asgar A.W., Fortier A., Pitre C., Bouabdallaoui N., Henri C., O’Meara E. (2022). Right ventricular function and its coupling to pulmonary circulation predicts exercise tolerance in systolic heart failure. ESC Heart Fail..

[34] Ghio S., Guazzi M., Scardovi A.B., Klersy C., Clemenza F., Carluccio E., Temporelli P.L., Rossi A., Faggiano P., Traversi E. (2017). Different correlates but similar prognostic implications for right ventricular dysfunction in heart failure patients with reduced or preserved ejection fraction. Eur. J. Heart Fail..

[35] de Pinto M., Coppi F., Spinella A., Pagnoni G., Morgante V., Macripo P., Boschini M., Guerra A.F., Tampieri F., Secchi O. (2024). The predictive role of the TAPSE/sPAP ratio for cardiovascular events and mortality in systemic sclerosis with pulmonary hypertension. Front. Cardiovasc. Med..

[36] Anastasiou V., Papazoglou A.S., Moysidis D.V., Daios S., Barmpagiannos K., Gossios T., Efthimiadis G.K., Karamitsos T., Ziakas A., Kamperidis V. (2024). The prognostic impact of right ventricular-pulmonary arterial coupling in heart failure: A systematic review and meta-analysis. Heart Fail. Rev..

[37] Vicenzi M., Caravita S., Rota I., Casella R., Deboeck G., Beretta L., Lombi A., Vachiery J.L. (2022). The added value of right ventricular function normalized for afterload to improve risk stratification of patients with pulmonary arterial hypertension. PLoS ONE.

[38] Sert S., Selcuk N., Yildirimturk O., Orhan G. (2022). Prognostic value of TAPSE/PASP ratio in right ventricular failure after left ventricular assist device implantation: Experience from a tertiary center. Turk. Gogus Kalp Damar Cerrahisi Derg..

[39] Ozpelit E., Akdeniz B., Ozpelit E.M., Tas S., Alpaslan E., Bozkurt S., Arslan A., Badak O. (2015). Impact of Severe Tricuspid Regurgitation on Accuracy of Echocardiographic Pulmonary Artery Systolic Pressure Estimation. Echocardiography.

[40] Fei B., Fan T., Zhao L., Pei X., Shu X., Fang X., Cheng L. (2017). Impact of severe tricuspid regurgitation on accuracy of systolic pulmonary arterial pressure measured by Doppler echocardiography: Analysis in an unselected patient population. Echocardiography.

